# Psychotropic drug-induced hyponatremia: results from a drug surveillance program–an update

**DOI:** 10.1007/s00702-021-02369-1

**Published:** 2021-07-01

**Authors:** Johanna Seifert, Martin Letmaier, Timo Greiner, Michael Schneider, Maximilian Deest, Christian K. Eberlein, Stefan Bleich, Renate Grohmann, Sermin Toto

**Affiliations:** 1 Department of Psychiatry, Social Psychiatry, and Psychotherapy, Hannover Medical School, Carl-Neuberg-Straße 1, 30625 Hannover, Germany; 2 Department of Medical Psychology and Psychotherapy, Medical University of Graz, Auenbruggerplatz 2, 8036 Graz, Austria; 3 Institute for Clinical Pharmacology of the Brandenburg Medical School, Immanuel Klinik Rüdersdorf, Seebad 82/83, 15562 Rüdersdorf, Germany; 4 Department of Psychiatry and Psychotherapy of the Brandenburg Medical School, Immanuel Klinik Rüdersdorf, Seebad 82/83, 15562 Rüdersdorf, Germany; 5 Department of Psychiatry and Psychotherapy, Ludwig Maximilian University of Munich, Nussbaumstr. 7, 80336 Munich, Germany

**Keywords:** Adverse drug reaction, Antidepressant drugs, Antiepileptic drugs, Antipsychotic drugs, Drug safety, Serum sodium concentration

## Abstract

Hyponatremia (HN) is the most common electrolyte imbalance (defined as a serum sodium concentration Na(S) of  < 130 mmol/l) and often induced by drugs including psychotropic drugs. AMSP (Arzneimittelsicherheit in der Psychiatrie) is a multicenter drug surveillance program that assesses severe or unusual adverse drug reactions (ADRs) occurring during treatment with psychotropic drugs. This study presents data from 462,661 psychiatric inpatients treated in participating hospitals between 1993 and 2016 and serves as an update of a previous contribution by Letmaier et al. (JAMA 15(6):739–748, 2012). A total of 210 cases of HN were observed affecting 0.05% of patients. 57.1% of cases presented symptomatically; 19.0% presented with severe symptoms (e.g., seizures, vomiting). HN occurred after a median of 7 days following the first dose or dose increase. Incidence of HN was highest among the two antiepileptic drugs oxcarbazepine (1.661% of patients treated) and carbamazepine (0.169%), followed by selective serotonin-norepinephrine reuptake inhibitors (SSNRIs, 0.088%) and selective serotonin reuptake inhibitors (0.071%). Antipsychotic drugs, tricyclic antidepressants, and mirtazapine exhibited a significantly lower incidence of HN. The risk of HN was 16–42 times higher among patients concomitantly treated with other potentially HN-inducing drugs such as diuretic drugs, angiotensin-converting-enzyme inhibitors, angiotensin II receptor blockers, and proton pump inhibitors. Female SSNRI-users aged  ≥ 65 years concomitantly using other HN-inducing drugs were the population subgroup with the highest risk of developing HN. The identification of high-risk drug combinations and vulnerable patient subgroups represents a significant step in the improvement of drug safety and facilitates the implementation of precautionary measures.

## Introduction

Hyponatremia (HN) is characterized by low serum sodium concentration (generally < 135 mmol/l) and is the most common type of electrolyte imbalance (Dineen et al. 2017). In this study, HN was set at serum sodium concentration Na(S) below 130 mmol/l, as it is more relevant to clinical practice (Spasovski et al. 2014; Mazhar et al. 2021). It is estimated that 15–20% of all patients admitted to the hospital suffer from this condition. The severity of symptoms is highly variable ranging from a complete absence of symptoms to mild symptoms such as headache, nausea, and imbalance, to serious symptoms such as seizures, cognitive impairment, and coma. The intensity of symptoms is associated with how quickly Na(S) decreases. A sudden onset drop (< 48 h) of Na(S) causes more dramatic clinical symptoms than a gradual decrease (Spasovski et al. 2014). The severe neurological symptoms of HN are caused by swelling of the brain cells resulting from redistribution of fluids from the extracellular to the intracellular compartment. These changes lead to an increase in intracranial pressure. When HN develops slowly over the course of several days or weeks, the body has more time to adapt and react to these changes resulting in a different array of major health complications. It is well-known that chronic HN is associated with a multitude of adverse health outcomes such as reduced cognitive functions, unsteadiness, falls (Renneboog et al. 2006), fractures, and osteoporosis (Gankam Kengne et al. 2008; Verbalis et al. 2010). Moreover, mild HN has proven to be an independent risk factor of death within the ambulatory setting (Gankam-Kengne et al. 2013).

Na(S) is managed by changes in the intake or output of water. While the specific underlying causes of HN are diverse, two main mechanisms result in low Na(S): water retention and—more frequently—loss of sodium. Depending on the underlying cause of HN, the circulating volume can be decreased, normal, or increased thus resulting in hypovolemic, euvolemic, or hypervolemic HN (Spasovski et al. 2014). Hypovolemic HN is caused by a depletion of extracellular fluid for example due to excessive sweating, vomiting, or most commonly, associated with the use of diuretic drugs (DIUs). Hypervolemic HN can be the result of severe illness such as liver cirrhosis, kidney disease, or congestive heart disease, all resulting in an increase in total body water (Dineen et al. 2017). These conditions may occur more frequently in psychiatric patients (Yip et al. 2020) or result in an increased risk of comorbid mental illness (Palmer et al. 2013). Moreover, and with particular relevance to psychiatric patients, hypervolemic HN can be caused by polydipsia, a condition which is most likely to occur in patients with schizophrenia (Dundas et al. 2007). Euvolemic HN is most often caused by the “syndrome of inappropriate ADH secretion” (SIADH) which is characterized by an increased release of ADH from the pituitary gland in absence of an appropriate stimulus. Among other possible causes such as pulmonary or malignant diseases, SIADH can be induced by drugs that chemically stimulate ADH secretion in the pituitary gland (Dineen et al. 2017).

Drug-induced HN is most commonly caused by DIUs, or more specifically, thiazide or thiazide-like diuretics (Liamis et al. 2008). While DIUs directly affect water and sodium homeostasis and result in renal loss of sodium, other drugs induce HN via one of three possible mechanisms: (1) central increase of ADH-secretion, (2) potentiation of the effects of endogenous ADH, or (3) lowering of the threshold for ADH secretion. Most psychotropic drugs associated with HN are believed to do so by inducing SIADH. A number of antidepressant drugs [ADDs, i.e., selective serotonin reuptake inhibitors (SSRIs), monoamine oxidase inhibitors (MAOIs), tricyclic antidepressants (TCAs)], antipsychotic drugs (APDs), and antiepileptic drugs (AEDs) are ascribed a certain risk for inducing HN (Liamis et al. 2008; Meulendijks et al. 2010; Falhammar et al. 2019a).

The present study aims to assess the risk of specific psychotropic drugs and drug combinations of inducing HN by using data from a 24-year timeframe collected by a large pharmacovigilance program. This study serves as an update to a previous publication by Letmaier et al. 2012 which analyzed 93 cases of HN detected during the time period 1993–2007.

## Methods

### The AMSP program

Founded in 1993, AMSP (German: “Arzneimittelsicherheit in der Psychiatrie”, “drug safety in psychiatry”) is an on-going pharmacovigilance program in German-speaking countries, which collects data on unusual and severe adverse drug reactions (ADRs) affecting all organ systems (e.g., psychiatric, neurological, cardiovascular) to psychotropic drugs occurring during inpatient treatment.

An ADR is considered severe if,

- it is (potentially) life-threatening or seriously endangers a patient’s health,
- it causes considerable impairment of everyday functioning, or
- it makes a transfer to another ward or department necessary for more specialized care (Grohmann et al. 2004, 2014).

The AMSP protocol provides additional guidelines based on each organ system to further standardize classification (Grohmann et al. 2014).

### Assessment and collection of ADRs

Data collection is performed by psychiatrists, who have been appointed as drug monitors. ADRs are either spontaneously reported by treating physicians or come to attention during questioning of treating physicians by drug monitors in regular intervals (i.e., at least bi-weekly) and are then documented on a standardized questionnaire. Information including age, sex, somatic and psychiatric diagnoses, all medication taken at the time of the ADR, as well as an exact description of the ADR including any relevant diagnostic procedures, are gathered. Each ADR is also assessed for risk factors specific to the patient, possible alternative explanations, previous occurrence of the same ADR, course of the ADR, and measures taken to counteract the ADR. A senior physician of the hospital then re-examines each documented ADR for plausibility. Selected cases of ADRs are presented and discussed at regional and national case conferences which are attended by drug monitors of participating hospitals, representatives of the national drug regulating authorities, and experts in drug safety. The probability of causal relationship between an adverse event and the drug(s) imputed is determined as follows:

Grade 1: possible (ADR unknown or alternative explanation more likely)

Grade 2: probable (ADR known for drug imputed and course of time and dose in accordance with previous experience; alternative explanation less likely)

Grade 3: definite (same as 2 with re-occurence of the ADR after re-exposure with the drug imputed)

Grade 4: questionable or not sufficiently documented.

More than one drug can be imputed for causing the ADR in question. In the case of HN, this can be either due to additive effects of the other drug(s) in causing SIADH or as a result of the direct action of a second drug on sodium homeostasis via mechanisms other than SIADH. When multiple drugs are held responsible for an ADR, the causal relationship of each drug is graded individually. In turn, AMSP distinguishes between three subgroups of ADR cases: cases in which only one drug was imputed, cases in which a combination of drugs was imputed, and ‘all cases’ which includes both of the previously mentioned cases (Grohmann et al. 2004).

In this study, all events of HN recorded by AMSP between 1993 and 2016 in which at least one psychotropic drug was imputed as ‘probable’ or ‘definite’ cause of HN are included. According to the different risk of HN of involved drugs observed in a prior evaluation of HN occurring during treatment with psychotropic drugs (Letmaier et al. 2012), causality was assessed as follows: SSRIs, selective serotonin-norepinephrine reuptake inhibitors (SSNRIs), TCAs, the two AEDs carbamazepine and oxcarbazepine, angiotensin II receptor blockers (ARBs), angiotensin-converting-enzyme inhibitors (ACE-Is), DIUs, proton pump inhibitors originally associated with hyponatremia (PPIHNs), and proton pump inhibitors not originally associated with hyponatremia (PPINNs) were imputed as probable whenever present; APDs, as well as MAOIs, mirtazapine, trazodone, and valproate, were imputed as only ‘possible’ contributors to HN, if no clear evidence for a ‘probable’ role (most relevant for this assessment was temporal relationship) was present as HN is a rare event under treatment with these drugs.

### Classification of psychotropic and non-psychotropic drugs

In the following, only psychotropic and non-psychotropic drugs involved in at least one case of HN are listed. Psychotropic drugs relevant to this study were classified as ADDs, APDs, and AEDs. ADDs were further classified as follows:

- SSRI: escitalopram, citalopram, sertraline, paroxetine, fluoxetine
- SSNRI: venlafaxine, duloxetine, milnacipran
- TCA: trimipramine, amitriptyline, doxepin
- Noradrenergic and specific serotonergic antidepressant (NaSSA): mirtazapine
- MAOI: tranylcypromine, moclobemide
- “other ADDs”: agomelatine, reboxetine, trazodone, vortioxetine

APDs were classified as “first generation antipsychotic drugs” (FGAs) or “second generation antipsychotic drugs” (SGAs). FGAs were sub-classified as “low potency” (lp) or “high potency” (hp).

- lp FGA: prothipendyl, melperone
- hp FGA: haloperidol, perazine, flupentixol, zuclopenthixol
- SGA: quetiapine, olanzapine, risperidone, aripiprazole, paliperidone palmitate

AEDs included carbamazepine, oxcarbazepine, and valproic acid.

Non-psychotropic drugs were classified as follows:

- ACE-I: ramipril, enalapril, quinapril, lisinopril, clizapril, captopril, imidapril
- ARB: valsartan, candesartan, olmesartan, losartan, irbesartan, telmisartan
- DIU: torasemide, hydrochlorothiazide, furosemide, amiloride, xipamide, triamterene, spironolactone, indapamide
- PPIHN: omeprazole, esomeprazole, lansoprazole
- PPINN: pantoprazole

### Demographic, illness-related, and drug use data

Data on drug use is gathered on two reference days per year on which all participating hospitals document all drugs prescribed on these days including exact dose, age, sex, psychiatric and somatic diagnoses. This information as well as a number of patients monitored each year and mean duration of inpatient stay allows an estimation of how many patients were exposed to a certain drug or combination of drugs. All obtained data is fully anonymized.

### Definition and categorization of HN

AMSP defines severe HN as a Na(S) < 130 mmol/l (Grohmann et al. 2004). As the collection of ADRs within the AMSP program is limited to severe ADRs and a Na(S) between 135 and 130 is defined as ‘mild’ and usually of lower clinical relevance (Spasovski et al. 2014), the Na(S) of < 130 mmol/l in this study is lower than the general definition of HN as determined by other studies (Mannesse et al. 2013; Mazhar et al. 2021). Cases with Na(S) ≥ 130 mmol/l were not eligible for inclusion. The following data only includes cases of HN in which the causal relationship of the drug was rated grade 2 or 3. In this study, each ADR presenting with HN was categorized according to severity of symptoms (Spasovski et al. 2014; Bullmann 2016):

(0) Absence of symptoms
(1) Mild symptoms: imbalance, slight confusion, lethargy, fatigue, edema, vertigo, paresthesia, loss of appetite
(2) Moderately severe symptoms: headache, nausea without vomiting, confusion
(3) Severe symptoms: vomiting, cardiorespiratory distress, seizures, delirium, coma, somnolence

### Statistical methods

Statistical analysis was performed using Excel^©^*.* Overall incidence of HN is given in percent and 95% confidence intervall (95% CI) of patients exposed to a specific drug, drug combination, drug class, or drug subclass. Because of the low actual incidence of HN and high overall exposure, the 95% CI was calculated using the exact method (Vollset 1993). Fisher’s exact tests were used to determine the significance of deviation between various drugs and drug combinations. A student’s t-test was used to compare the means of two groups. The level of significance was set at *p* < 0.05.

*Risk of HN during polypharmacy/concomitant drug use*—Risk of HN under treatment with certain drug/drug class combinations (i.e., SSRIs, SSNRIs, carbamazepine) was analyzed for specific drug combinations in which the psychotropic drug/drug class showed sufficiently high rates of HN both when imputed alone as well as in combination with other drugs. SSNRIs and SSRIs were subsumed as a group—the two SSNRIs venlafaxine and duloxetine both had similar rates of HN, whereas a class-effect has been described for SSRIs (Egger et al. 2006; De Picker et al. 2014). Further, carbamazepine as an individual drug was selected because, though chemically related to oxcarbazepine, the two AEDs show significantly different risks of HN (more than tenfold). Clinically relevant drug combinations were determined according to the number of patients treated with the respective drug combination (i.e., ≥ 1,900 patients) as drug combinations used in < 1,900 patients resulted in increasingly wider 95% CIs limiting our ability to draw valid conclusions. Due to the insufficient number of patients treated with oxcarbazepine in combination with other drugs, drug combinations with oxcarbazepine are not further analyzed.

*Risk of HN according to age and sex*—SSRIs and SSNRIs were selected for analysis according to age and sex because they were the drug classes most commonly used both in the treatment of patients < 65 and ≥ 65 years of age. The analysis of other drugs/drug groups (e.g., carbamazepine) in this manner resulted in very wide, and therefore imprecise, 95% CIs.

### Ethics review

Analyses using the AMSP database have been approved by the Ethics Committee of the University of Munich and the Ethics Committee of the Hannover Medical School (Nr. 8100_BO_S_2018). This study adheres to the Declaration of Helsinki and its later amendments. The AMSP programme is a continuous observational post-marketing drug surveillance programme and does not interfere with the ongoing clinical treatment of the patients under surveillance.

## Results

### Demographic and illness-related data

A total of 495,615 psychiatric inpatients were monitored within the hospitals participating in the AMSP project between 1993 and 2016. 93.4% of patients (462,661 patients) were treated with at least one psychotropic drug. A total of 210 cases of HN affecting 0.05% of patients treated with psychotropic drugs were documented during this time period that fulfilled AMSP’s criteria. Females were affected in 156 (74.3%) of cases. While patients aged ≥ 65 years constituted about a fifth of the total study population, nearly half of patients with HN were ≥ 65 years of age indicating that patients within this age group were nearly 3 times more likely to develop HN than younger patients (< 65 years; 0.104% vs. 0.290%, *p* < 0.001). More than half (i.e., 58.1%) of patients with drug-induced HN suffered from mood disorders (ICD-10: F3). Patients suffering from substance-related disorders (ICD-10: F1) presented the highest incidence (0.111% of patients within this subgroup) of HN within diagnostic subgroups (Table 1).

**Table 1:** Characteristics of the study population

	All patients monitored, N (% of 462,661 patients)	Patients with HN, N (% of 210 patients)	% of patients with HN	*p* value
All patients	462,661 (100)	210 (100)	**0.05**	
Diagnosis (ICD-10)				
Organic disorders (F0)	56,419 (12.2)	19 (9.0)	0.034	*χ* ^2^ = 74.651; df = 4; *p* < 0.001*
Substance-related disorders (F1)	20,637 (4.5)	23 (11.0)	0.111	
Schizophrenia (F2)	158,037 (34.2)	30 (14.3)	0.019	
Mood disorders (F3)	171,165 (37.0)	122 (58.1)	0.010	
Others (F4–F9)	56,403 (12.1)	16 (7.6)	0.028	
Age of patients treated with psychotropic drugs				
< 65 years	363,562 (78.6)	107 (51.0)	0.029	*χ*^2^ = 95.153; df = 1; *p* < 0.001*
≥ 65 years	99,099 (21.4)	103 (49.0)	0.104	
Sex				
Male	204,071 (44.1)	54 (25.7)	0.026	*χ* ^2^ = 28.808; df = 1; *p* < 0.001*
Female	258,590 (55.9)	156 (74.3)	0.060	

*N* number, *HN* hyponatremia, *df* degrees of freedom, *ICD-10* International Classification of Disease, 10th Version

*statistically significant

### Serum sodium concentration and symptom presentation

Most frequently patients presented without any clinical manifestations of HN (57.1%); 19.0% showed severe symptoms (Table 2). The mean (± standard deviation) Na(S) among all patients with drug-induced HN was 120.5 ± 6.0 mmol/l (range 102.0–129.0 mmol/l, median 119.0 mmol/l). Patients presenting without any symptoms had higher Na(S) (123.0 ± 4.3 mmol/l) than patients suffering from mild to severe symptoms (117.0 ± 6.1 mmol/l; *p* < 0.001). Na(S) differed significantly between cases of moderate HN compared to severe HN (*p* = 0.006), while this was not the case for the comparison of moderate HN compared to mild symptomatic HN (*p* > 0.05). Table 2 also shows the mean and median Na(S) in correlation to clinical symptoms as well as several symptoms of severe HN (e.g., seizures, delirium, vomiting, falls, somnolence, coma). In cases of severe HN, Na(S) ranged from a minimum of 102.0 mmol/l to 128.0 mmol/l. Of note, Na(S) was only mildly lowered (i.e., 125.0 mmol/l) in some cases presenting with severe symptoms, while on the other hand, cases with a minimum Na(S) of 109.0 mmol/l did not show any clinical symptoms.

**Table 2:** Clinical presentation of drug-induced hyponatremia according to severity, symptoms, and serum sodium concentration

Clinical presentation of HN	Number of cases (%)	Mean Na(S) ± SD (in mmol/l)	Median Na(S) in mmol/l	Range; Min–Max (in mmol/l)
All cases	210 (100)	120.5 ± 5.6	119.0	102–129
Asymptomatic	120 (57.1)	123.0 ± 4.3	124.0	109–129
All symptomatic cases	90 (42.9)	117.0 ± 6.1	118.0	102–129
Mild symptoms	23 (11.0)	121.6 ± 4.1	122.0	117–128
Moderately severe symptoms	22 (10.5)	119.4 ± 4.8	118.5	111–129
Severe symptoms	40 (19.0)	115.4 ± 6.2	116.0	102–128
Seizures	16	115.1 ± 6.1	114.5	104–123
Delirium	17	115.2 ± 5.5	116.0	104–123
Vomiting	14	114.5 ± 6.2	116.5	102–125
Falls	14	114.5 ± 6.5	116.5	102–125
Somnolence	8	113.1 ± 6.1	114.5	102–120
Coma	2	104.0 ± 0	104.0	104

*HN* hyponatremia; *Na(S)*: serum sodium concentration; *SD*: standard deviation; *min*: minimum; *max*: maximum

### Psychotropic drugs associated with HN

HN was caused by a single psychotropic drug in only 61 cases (29.0%). The majority (i.e., 71.0%) of cases were caused by more than one (psychotropic) drug. Table 3 shows the psychotropic drug classes/drugs involved in HN. Unless explicitly stated otherwise, the following refers to all cases of HN (i.e., multiple and single imputations).

**Table 3:** Incidence of drug-induced hyponatremia among psychotropic drugs and drug classes

Drug class/drug	N patients exposed	N patients with HN (all imputations)	N cases of HN with single imputation	% of patients with HN (all imputations)	95% CI
Antidepressant drugs					
Any ADD	243,588	124	23	0.051	0.042–0.061
SSRI	92,496	66	15	0.071	0.055–0.091
Citalopram	24,904	30	6	0.120	0.081–0.172
Escitalopram	25,667	17	3	0.066	0.039–0.106
Sertraline	21,868	15	5	0.069	0.038–0.113
Paroxetine	10,298	3	1	0.029	0.008–0.065
Fluoxetine	5849	1	0	0.172	0.000–0.095
SSNRI	56,530	50	4	0.088	0.066–0.123
Venlafaxine	41,559	37	2	0.089	0.063–0.123
Duloxetine	14,343	12	2	0.084	0.043–0.146
Milnacipran	858	1	0	0.117	0.003–0.648
NaSSA	63,182	1	0	0.002	0.003–0.021
Mirtazapine	60,305	1	0	0.002	0.000–0.009
TCA	56,666	9	4	0.016	0.007–0.030
Amitriptyline	14,089	6	3	0.043	0.016–0.093
Clomipramine	6174	2	0	0.032	0.004–0.117
Trimipramine	13,604	1	1	0.007	0.000–0.041
MAOI	4855	2	0	0.041	0.005–0.149
Tranylcypromine	2384	1	0	0.042	0.001–0.233
Moclobemide	2471	1	0	0.040	0.001–0.225
other ADDs	24,761	5	0	0.020	0.007–0.047
Trazodone	12,571	4	0	0.032	0.009–0.081
Vortioxetine	501	1	0	0.200	0.005–1.107
Antiepileptic drugs					
any AED	99,846	89	35	0.089	0.072–0.110
Carbamazepine	24,308	41	15	0.169	0.121–0.229
Oxcarbazepine	2710	45	19	1.661	1.214–2.216
Valproic acid	42,259	7	1	0.017	0.007–0.034
Antipsychotic drugs					
any APD	333,175	16	3	0.005	0.003–0.008
hp FGA	92,980	6	0	0.006	0.002–0.014
Haloperidol	37,650	3	0	0.008	0.002–0.023
Perazine	15,495	2	2	0.013	0.002–0.047
Zuclopenthixol	7144	1	0	0.014	0.000–0.078
lp FGA	102,468	2	0	0.002	0.000–0.007
Melperone	18,984	1	0	0.005	0.000–0.029
Prothipendyl	15,742	1	0	0.006	0.000–0.035
SGA	226,161	11	1	0.005	0.002–0.009
Olanzapine	54,822	2	0	0.004	0.000–0.013
Quetiapine	66,209	3	0	0.005	0.001–0.013
Aripiprazole	15,988	1	0	0.006	0.000–0.035
Risperidone	51,683	5	0	0.010	0.003–0.023
Paliperidone palmitate	1279	1	1	0.078	0.002–0.435

This table only includes cases in which the psychotropic drug was considered a ‘probable’ or ‘definite’ cause of HN; ‘possible’ causality is not included. Psychotropic drugs never imputed in HN were benperidol, bromperidol, haloperidol decanoate, flupentixol, fluphenazine, pipamperone, flupentixol, chlorprothixene, zotepine, clotiapine, ziprasidone, sertindole, levomepromazine, perphenazine, promethazine, promazine, amisulpride, agomelatine, mianserin, maprotiline, nefazodone, reboxetine, imipramine, nortriptyline, doxepin, bupropion, fluvoxamine, topiramate, opipramol, diazepam, lorazepam, zopiclone, zolpidem, chloral hydrate, clomethiazole, pregabalin, lamotrigine, biperiden, memantine, donepezil, galantamine, rivastigmine, and methylphenidate.

*N* number, *HN* hyponatremia, *95% CI* 95% confidence interval, *ADD* antidepressant drug, *APD* antipsychotic drug, *AED* antiepileptic drug, *TCA* tricyclic antidepressant, *SSRI* selective serotonin reuptake inhibitor, *SSNRI* selective serotonin-norepinephrine reuptake inhibitor, *NaSSA* noradrenergic and specific serotonergic antidepressant, *MAOI* monoamine oxidase inhibitor, *FGA* first generation antipsychotic drug, *SGA* second generation antipsychotic drug, *hp* high potency, *lp* low potency

*Antidepressant drugs*: out of all cases of HN, ADDs were the psychotropic drug class most commonly imputed in psychotropic drug-induced HN (124 cases, 59.0% of HN cases) – 0.051% of patients treated with ADDs developed HN. SSNRIs were the ADD-subgroup with the highest risk of HN affecting 0.088% of SSNRI-users, while citalopram was the individual drug most commonly associated with HN (0.120% of patients exposed). Only a single probable case was observed with the imputation of NaSSAs, therefore resulting in the lowest risk of HN among ADDs. In about one-fifth of cases (23 cases of HN, 18.5% of ADD-induced HN), an ADD was imputed alone for causing HN–SSRIs in 15, SSNRIs in 4, and TCAs in 4 cases; MAOIs and NaSSAs were never imputed alone.

*Antiepileptic drugs*: AEDs were imputed in 89 cases of HN (42.4% of HN cases) and were the psychotropic drug class with the highest incidence of HN (0.089% of patients exposed) and also most likely to be imputed alone (35 cases, 39.3% of AED-induced HN). Oxcarbazepine showed the by far highest risk of HN affecting 1.661% of patients treated. Oxcarbazepine was imputed alone in 19 cases of HN (1.59% of patients treated with oxcarbazepine alone; 95% CI 0.96–2.48; data not shown). Carbamazepine showed the second highest risk of HN among all psychotropic drugs (0.169%).

*Antipsychotic drugs*: in relation to the high number of exposed patients, APDs were only very rarely associated as ‘probable’ or 'definite' cause of HN. Overall, 16 such cases of APD-induced HN were detected (7.6% of HN cases) of which 4 occurred without imputation of other drugs under treatment with perazine (2 cases), paliperidone palmitate (1 case), and a combination of 3 APDs (zuclopenthixol + aripiprazole + risperidone). Including the cases with ‘possible’ imputations of an APD, the risk of drug-induced HN remained low (single and multiple imputation: 115 cases, 0.03%; imputed alone: 6 cases, 0.002%; data not shown in tables/figures).

### Dose-dependent effects of HN

Among cases of HN in which a single drug was imputed, dose-dependent effects were found exclusively for oxcarbazepine (mean dose of all patients exposed (MD_all_): 865.0 ± 482.0 mg vs. mean dose of patients with HN (MD_HN_): 1129.0 ± 436.3; *p* = 0.017).

When imputed alone, SSRIs and SSNRIs did not exhibit dose-dependency. However, when considering all cases of HN including multiple imputations, mean dosage of several drugs was significantly lower in patients who experienced HN compared to all patients exposed. This was observed for sertraline (MD_all_: 98.0 ± 56.7 mg vs. MD_HN_: 64.3 ± 37.1 mg; *p* = 0.021), venlafaxine (MD_all_: 190.8 ± 85.4 vs. MD_HN_: 137.2 ± 59.6 mg; *p* < 0.001), and duloxetine (MD_all_: 80.7 ± 38.9 mg vs. MD_HN_: 52.5 ± 21.7 mg; *p* = 0.001).

### Polypharmacy and concomitant drug use

**Fig. 1:**
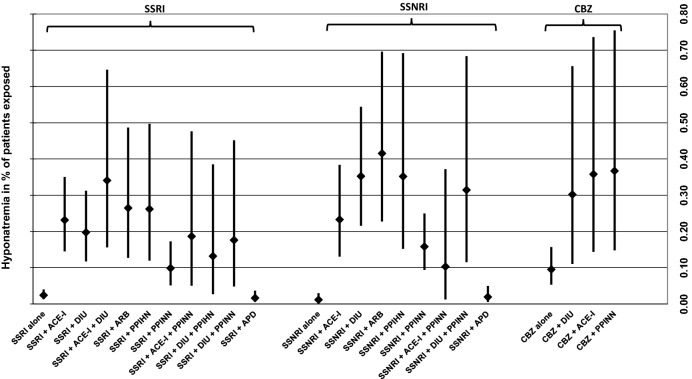
Incidence of hyponatremia including 95% CI of SSRIs, SSNRIs, and CBZ alone and in combination with other drugs. Only drug combinations used in ≥ 1900 patients are depicted. 95% CI 95% confidence interval, SSRI selective serotonin reuptake inhibitor, SSNRI selective serotonin-norepinephrine reuptake inhibitor, CBZ carbamazepine, APD antipsychotic drug, ACE-I angiotensin-converting-enzyme inhibitor, ARB angiotensin II receptor blocker, DIU diuretic drug, PPIHN proton pump inhibitor originally associated with hyponatremia, PPINN proton pump inhibitor not originally associated with hyponatremia

Figure 1 shows the most common combinations of psychotropic drug groups and individual drugs (i.e., SSRIs, SSNRIs, carbamazepine) with other HN-inducing drugs involved in HN. Risk of HN increased when the respective psychotropic drug class or drug was combined with other potentially HN-inducing drugs used to treat internal illnesses such as ACE-Is, ARBs, DIUs, PPIHNs, and PPINNs. For example, when combined with a DIU or ACE-I, SSRI-users had a tenfold higher risk of developing HN than those treated without DIUs or ACE-Is. The risk of HN increased further when an SSRI-user was treated with both a DIU and an ACE-I. The same was observed among SSNRI-users. Similarly, the risk of HN increased among carbamazepine-users when used in combination with DIUs, ACE-Is, or PPINNs. It must be noted that confidence intervals—especially of drug combinations—are often wide and overlapping, therefore, disabling precise estimations.

The concomitant use of any PPI was also associated with a higher incidence of HN—concomitant use of a PPIHN was more likely to induce HN than the use of PPINNs. In fact, when used in combination with a PPIHN, patients treated with SSRIs and SSNRIs were more than twice as likely to develop HN than when these drugs were used with the PPINN pantoprazole.

The risk of HN did not significantly increase when SSRIs or SSNRIs were used in combination with an APD. Even when all cases with a ‘possible’ co-imputation of APDs were included in the analysis, incidence was comparable to that of ADDs (0.01%, 95% CI 0.01–0.02 for ADD + APD; data not shown), SSRIs (0.02%, 95% CI 0.01–0.03 for SSRI + APD, Fig. 1), or SSNRIs (0.02%, 95% CI 0.00–0.05 for SSNRI + APD, Fig. 1) when imputed alone.

A combination of drugs was imputed in 36 of the HN cases with severe symptoms detected in this study. A single psychotropic drug was imputed alone in only 4 cases (one case each under treatment with oxcarbazepine, carbamazepine, sertraline, and citalopram; data not shown in tables/figures).

### Polypharmacy and concomitant drug use according to gender and age

**Fig. 2:**
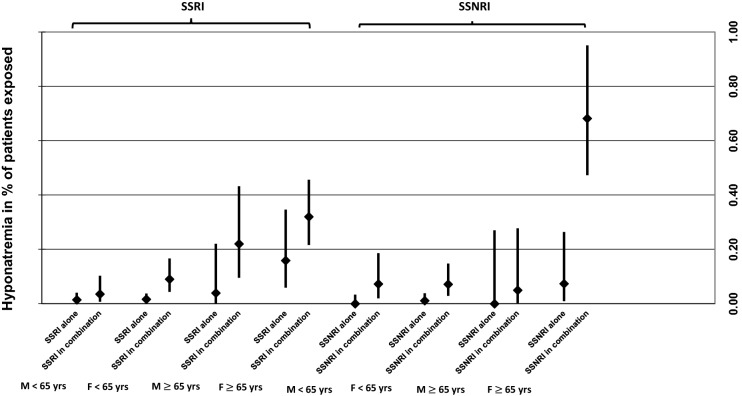
Incidence of hyponatremia including 95% CI for SSRI- and SSNRI-users according to sex, age, and concomitant drug use. 95% CI 95% confidence interval, M males, F females, Yrs years, SSRI selective serotonin reuptake inhibitor, SSNRI selective serotonin-norepinephrine reuptake inhibitor

Figure 2 shows frequencies of HN in SSRI- and SSNRI-users according to gender and age. Female SSNRI-users aged ≥ 65 years concomitantly using other HN-inducing drugs were the subgroup with the highest risk of developing HN—0.68% (95% CI 0.47–0.95) of patients within this group developed HN. The risk of HN was significantly higher among this patient subgroup when compared to males of the same age group (*p* = 0.02). With confidence intervals overlapping among the other age and sex groups of SSRI- and SSNRI-users, no such marked differences between cases in which an SSRI/SSNRI was imputed alone vs. SSRI/SSNRI was imputed in combination with other drugs was detected.

### Time to onset of HN

In cases in which a single psychotropic drug was imputed, HN occurred after a median of 7 days (range 1–2111 days; interquartile range 15.5 days) after initiation of the imputed drug or increase of dosage. In 40 cases (64.5%), dosage of the imputed psychotropic drug had been increased prior to the detection of HN. When imputed alone, HN occurred within 3 weeks in 87% of cases imputing SSRIs, in 75% of cases imputing SSNRIs, in 66% of cases imputing carbamazepine, and in 83% of cases imputing oxcarbazepine.

When multiple drugs were held responsible for HN, SSRIs, SSNRIs, carbamazepine, and oxcarbazepine had been used for ≤ 3 weeks in more than 50% of cases. The co-imputed non-psychotropic drugs had been used for a longer period of time in a majority of cases (i.e., for more than 3 weeks in about 2/3 of co-imputations for PPIHNs, in > 80% for ACE-Is, ARBs, PPINNs, and DIUs, and for > 3 months in 52% of co-imputations of PPIHNs and > 63% of co-imputations of ACE-Is, ARBs, PPINNs, and DIUs).

### Risk factors

In 153 cases (72.7%), no risk factors for the occurrence of HN were identified. However, under consideration of patients with severe symptomatic HN (e.g., seizure, coma, delirium, somnolence; *n* = 40), predisposing risk factors for HN or for the development of severe symptoms were identified in more than half of the patients affected (24 cases; 60.0%) such as substance use disorders (6 cases), gastrointestinal disturbances prior to HN (i.e., vomiting/diarrhea; 4 cases), low Na(S) prior to drug initiation (4 cases), or polydipsia (3 cases). Among patients presenting with a seizure, 3 patients had experienced at least one previous seizure and 6 suffered from pre-existing brain damage.

### Countermeasures and course of HN

In most cases, one or more of the imputed drugs was discontinued (184 cases; 87.6%). Daily dose was reduced in 22 cases (10.4%), while treatment was continued without any alteration in only a single case of asymptomatic HN (0.5%). 49 patients (23.3%) required a transfer to an internal/neurological department or intensive care unit to receive more specialized care. Four cases resulted in life-threatening symptoms (i.e., cerebral edema—2 cases, aspiration pneumonia resulting from a seizure, and central pontine myelinolysis after rapid sodium substitution—1 case each).

Pharmacological countermeasures (i.e., intravenous hypertonic or isotonic saline, sodium tablets) were taken in 131 cases (62.4%); non-pharmacological measures (i.e., high-sodium diet, fluid restriction) were taken in 40 cases (19.0%).

At the end of the observation period, HN had fully subsided in a majority of cases (166 cases; 79.1%) or was in the process of subsiding (30 cases; 14.3%). In 11 cases (5.2%), HN remained unchanged, while 1 case resulted in permanent damage following coma and intracranial hypertension (0.5%). The course of HN was unknown in 2 cases (1.0%).

## Discussion

This study examined the incidence of HN occurring under treatment with psychotropic drugs. Oxcarbazepine was the psychotropic drug with the greatest risk of HN affecting 1.66% of patients treated, followed by carbamazepine, SSNRIs, and SSRIs. Combination treatments, especially with DIUs, ACE-Is, and ARBs, were found to significantly increase the risk of developing HN by up to a 40-fold. A smaller number of cases of HN were associated with the use of other psychotropic drugs (i.e., mirtazapine, TCAs, and APDs). HN was detected a median of 7 days after initiation of treatment or increase of dosage of the imputed psychotropic drug.

### HN under treatment with psychotropic drugs

Despite the high risk of HN reported by some authors (Strachan and Shepherd 1998), many cases of psychotropic-drug induced HN may present asymptomatically or with unspecific symptoms—as was the case in the present study—which in turn may mean, that these cases remain unnoticed if Na(S) is not monitored regularly. This may have contributed to an under-reporting of HN in this study. Patients with ‘asymptomatic’ HN in this study presented with a mean Na(S) of 124 mmol/l, a Na(S) generally expected to be associated with moderate to severe HN (Spasovski et al. 2014). As HN often presents with nonspecific symptoms such as lethargy, fatigue, and confusion, which can easily be mistaken for worsening of depressive symptoms, these cases may have wrongfully been deemed ‘asymptomatic’. In the present study assessing only severe ADRs, HN was defined as Na(S) < 130 mmol/l while other authors defined HN as Na(S) of < 135 mmol/l. This discrepancy in definition may contribute to the wide range of frequencies of psychotropic drug-induced HN reported by other authors. Moreover, due to AMSP’s strict criteria and the inclusion of cases only if Na(S) < 130 mmol/l, it is likely that many more patients suffered from HN as defined by other authors.

### Antidepressant drugs

An association between SSRIs and the occurrence of HN was first noted by Hwang and Magraw (1989) and has been described for all currently marketed SSRIs (Jacob and Spinler 2006). While ADD-induced HN is most commonly associated with the use of SSRIs and SSNRIs, it is not an ADR specific to these classes of ADDs (Wright and Schroeter 2008). In general, HN is a phenomenon which has been described to occur under the use of almost all ADDs (De Picker et al. 2014). TCAs and mirtazapine have shown a lower risk of HN in comparison to SSRIs or SSNRIs (Farmand et al. 2018), as was the case in the present study. A single case of HN with co-imputation of mirtazapine was documented by AMSP. Other authors disagree stating mirtazapine has a higher risk of causing HN than SSRIs and SSNRIs (Mazhar et al. 2019).

It is still unclear if any single SSRI has a higher risk of HN than the others so that in general a class-effect of SSRIs is postulated (Egger et al. 2006; De Picker et al. 2014). One study found that up to 24% of hospitalized patients treated with either of the two SSRIs paroxetine or fluoxetine developed HN at some point during hospitalization (Strachan and Shepherd 1998). While other SSRIs were not examined in the above-mentioned study, therefore impeding further comparisons to the present study, it is of note that paroxetine was found to have the lowest risk of HN among SSRIs in this study. Other studies agree with the present results that escitalopram and citalopram are associated with higher incidences of HN compared to other substances within the drug class (Degner et al. 2004; Coupland et al. 2011a, 2011b; Letmaier et al. 2012; Shepshelovich et al. 2017), whereas paroxetine seems to less frequently cause HN (Letmaier et al. 2012). This seemingly lower rate of paroxetine- and fluoxetine-induced HN may derive from a bias in the use of paroxetine and fluoxetine. Paroxetine is a potent inhibitor of the cytochrome P450 isoenzyme 2D6 potentially causing significant drug–drug interactions (Bahar et al. 2018) and therefore may show higher utilization among younger patients who are less likely to be treated with multiple drugs and less predisposed to develop HN. Similarly, as fluoxetine is the SSRI with the longest half-life, which may be even longer in older patients potentially resulting in an increased risk for drug–drug interactions (Ferguson and Hill 2006), use of fluoxetine may also be lower among older patients.

SSRIs cause serotonin levels to increase, which leads to the stimulation of 5-HT_1c_ and 5-HT_2_ receptors in turn activating ADH secretion (Spigset and Hedenmalm 1995). Some authors have hypothesized that the risk of an ADD to cause HN correlates with the drugs’ potency to inhibit the re-uptake of serotonin (Degner et al. 2004), which may in part explain the higher incidence of HN among users of citalopram and escitalopram. Further, it is postulated that norepinephrine stimulates the secretion of ADH (Knigge et al. 1999), providing a possible explanation for the slightly higher risk of HN under treatment with SSNRIs (0.09%; imputed alone and in combination) than with SSRIs (0.07%) found in the present study. Overall, the incidence of HN among SSNRIs is less researched than among SSRIs. Studies examining the risk of HN under treatment with venlafaxine found risks equal to or higher than for SSRIs, while duloxetine is less examined (De Picker et al. 2014). The present study confirms these results.

### Antiepileptic drugs

Several AEDs are attributed a high risk of inducing HN. Carbamazepine (Shepshelovich et al. 2017; Falhammar et al. 2018) and, even more frequently, oxcarbazepine have been found to be the AEDs most likely to cause HN (Liamis et al. 2008; Falhammar et al. 2018). In fact, HN has been described as the leading ADR in psychiatric inpatients during treatment with oxcarbazepine (Druschky et al. 2018). Intravooth et al. detected HN in 43% of oxcarbazepine-users and 33% of oxcarbazepine-users in a cohort consisting of 560 patients, though none of these cases were classified as severe (2018). The present study detected the highest incidence of HN in oxcarbazepine-users (1.66%) in comparison to all other psychotropic drugs, followed by carbamazepine (0.17%). Even when imputed alone, oxcarbazepine had a nearly 16-fold higher risk of HN than carbamazepine and an almost 80-fold increased risk in comparison to SSRIs alone.

Further, this study detected 7 events of HN associated with the use of valproic acid. The risk of HN during treatment with other AEDs such as valproic acid (Druschky et al. 2018; Intravooth et al. 2018) or levetiracetam (Intravooth et al. 2018) are only rarely associated with the occurrence of HN. However, this study found valproic acid to have a comparable risk of HN (0.02%) as the SSRI paroxetine (0.03%).

### Antipsychotic drugs

The role of APDs in inducing HN is less conclusive than that of the above-mentioned drug groups (Meulendijks et al. 2010). First reports of HN occurring under treatment with APDs arose in the 1970s, when thiothixene (a thioxanthene derivate) (Ajlouni et al. 1974) and haloperidol (Peck and Shenkman 1979) were found to impair the patient’s ability to excrete a free water load. Patients treated with FGAs appear to be more likely to experience HN than those treated with SGAs (Yang and Cheng 2017; Falhammar et al. 2019a). A recent case–control study demonstrated that the use of any FGA was more likely to cause hospitalization due to HN upon initiation of drug treatment in comparison to SGAs. When considering ongoing treatment, the risk of HN decreased for FGAs while it slightly increased for SGAs (Falhammar et al. 2019a). However, in comparison to non-users, SGA-users may have an increased risk of hospitalization due to HN (Gandhi et al. 2016). In many of the published reports of APD-induced HN, causality of the APD was deemed ‘possible’ and to a lesser extent ‘probable’. There are, however, also reports of rechallenge and subsequent reoccurrence of HN for several APDs including haloperidol, quetiapine, and aripiprazole (Meulendijks et al. 2010).

The most recent study assessing the risk of HN under treatment with APDs examined the relationship between receptor occupancy of APDs and HN. While the degree of occupancy of dopamine D3 receptors positively correlated with the occurrence of HN, a negative association was found for the occupancy of serotonin 5-HT_2A_ receptors. Mazhar et al., therefore, concluded that APD-induced HN may be caused by an unbalanced inhibition of dopamine D3 and serotonin 5-HT_2A_. Among the APDs included in Mazhar et al.’s study with the highest degree of disproportionality—and therefore higher risk for HN—were amisulpride, flupentixol, risperidone, and olanzapine (2021). While the present study also observed HN under treatment with risperidone and olanzapine, flupentixol and amisulpride were not associated with the occurrence of drug-induced HN.

The present study detected a total of 16 events of HN probably or definitely associated with APDs; APDs (as a single APD or combination of multiple APDs) were imputed exclusively in only 4 cases. While this very low number of events does not allow a robust conclusion to be drawn, the present study found a slightly higher incidence of APD-induced HN than the study by Letmaier et al. (Letmaier et al. 2012) (0.005% vs. 0.003%). In relation to their utilization rates, lp FGAs exhibited a slightly lower risk of HN than hp FGAs and SGAs.

### Concomitant drug use and HN

When used in combination with other potentially HN-inducing drugs such as DIUs (Liamis et al. 2008), ARBs, ACE-Is (Falhammar et al. 2020), and PPIs (Falhammar et al. 2019b), the incidence of HN increased dramatically among SSRI-, SSNRI-, and carbamazepine-users. Affecting 0.42% of patients, SSNRI + ARB was the drug combination (used in ≥ 1,900 patients) with the highest risk of HN, followed by SSNRI + DIU and SSNRI + PPIHN (0.35% each). The concomitant prescription of psychotropic drugs with a thiazide or thiazide-like diuretic is a well-described risk factor for severe HN through possibly complementary mechanisms (Rosner 2004; Kim et al. 2014). The use of thiazides and thiazide-like diuretics has previously been shown to increase the risk of HN in patients treated with psychotropic drugs by a fourfold (odds ratio (OR) = 4.04, 95% CI = 1.03–15.70) (Mannesse et al. 2013), an OR which is substantially exceeded in the present study. Due to the larger study sample and higher number of registered HNs, this study provides narrower, and therefore more accurate confidence intervals for several drug combinations in comparison to the previous study by Letmaier et al. SSRI-users with concomitant use of DIUs had a tenfold higher risk for HN, which is also higher than the risk determined by Letmaier et al. (sevenfold) (Letmaier et al. 2012). SSNRI-users concomitantly using DIUs had a 35 times greater risk that those not using DIUs in this study. In fact, incidence of HN in SSNRI-users without other potentially HN-inducing dugs was minimal (0.01%) and increased significantly (16-fold to 42-fold) in combination with ACE-Is, DIUs, ARBs, PPIHNs, and PPINNs. This finding implicates that SSNRIs have a particularly high potential of exerting additive pharmacodynamic effects when used with other drugs. Moreover, in most cases presenting with severe symptoms of HN in this study (i.e., 36 out of 40 cases), multiple drugs were considered to be causally associated with the occurrence of HN. This finding indicates that not only is HN more likely to occur when multiple HN-inducing drugs are combined, but the risk of severe symptoms is also increased.

On the other hand, it has been demonstrated that the prescription of more than one potentially SIADH-inducing medication led to a higher overall prevalence of HN but did not impact the severity of symptoms. This finding may suggest that there are no synergistic effects between SIADH-inducing drugs (Shepshelovich et al. 2017). This may explain why the combination SSRI/SSNRI + APD did not lead to an increased risk of HN. Similarly, concomitant use of SSRIs and carbamazepine has been found not to be related to the occurrence of HN under treatment with APDs (Yang and Cheng 2017). In our study, concomitant use of APDs did not increase the risk of HN in SSRI and SSNRI-users.

### Use of PPIHN versus PPINN

PPIs are often considered devoid of ADRs (Nachnani et al. 2015) and are liberally prescribed (Falhammar et al. 2019b). Most published case reports of PPI-induced HN were related to the use of omeprazole and esomeprazole (Ferreira et al. 2016; van der Zalm et al. 2020) which are also viewed as the PPIs with the highest risk for this overall rare PPI-induced ADR (Falhammar et al. 2019b). Lansoprazole was first reported to be involved in HN in 2000 (Fort et al. 2000), while indications of pantoprazole-induced HN arose in 2014 (Naharcý et al. 2014). In this study, PPIs are categorized as PPIHNs (i.e., omeprazole, esomeprazole, lansoprazole) or PPINNs (i.e., pantoprazole) reflecting these temporal aspects, an approach which was not previously used by Letmaier et al. (2012) as pantoprazole was still considered to be devoid of HN as an ADR at that time. This study demonstrates that the combined use of PPIHNs with SSRIs and SSNRIs each showed a higher risk of HN than when used with the PPINN pantoprazole. In fact, the risk of HN was more than twice as high among patients treated with PPIHN + SSRI than those treated with PPINN + SSRI while this discrepancy was not as pronounced among SSNRI users. These clinically relevant implications are useful in the selection of a safe drug regimen, especially when treating patients with other risk factors for HN (see below).

### Dosage and time to onset of drug-induced HN

As found in the present study, risk of drug-induced HN appears to be the highest during the first 2–3 weeks of treatment (Liu et al. 1996; Wilkinson et al. 1999; Kirby and Ames 2001; Madhusoodanan et al. 2002; Fabian et al. 2004; Mazhar et al. 2020). About 75% of patients who suffer from SSRI-induced HN develop HN within the first 30 days of treatment. However, HN can also occur after long-term treatment (i.e., several years) (Meulendijks et al. 2010), as was also the case here.

This study found that in cases of multiple imputations, the imputed non-psychotropic drugs had often been administered for more than 3 weeks or even for more than 3 months. Due to the psychiatric inpatient setting, it is expected that psychotropic medication is more frequently adjusted than non-psychotropic medication. This consideration indicates that previously well-tolerated potentially HN-inducing drugs are more likely to cause HN in combination with recently added potentially HN-inducing psychotropic drugs. Further supporting this is that lower doses of sertraline, venlafaxine, and duloxetine were used in the treatment of patients in this study who experienced HN due to combinations of multiple drugs.

The occurrence of SSRI-induced HN appears to be unrelated to dose (Madhusoodanan et al. 2002; Egger et al. 2006) or plasma levels (Stedman et al. 2002; Fabian et al. 2004). Dose-dependent effects of HN were found only for oxcarbazepine in this study. While this is also reflected in literature (Lin et al. 2010; Kim et al. 2014), the present study was unable to validate the well-established dose-dependent effects described for carbamazepine (Van Amelsvoort et al. 1994; Kuz and Manssourian 2005; Holtschmidt-Täschner and Soyka 2007). If this is the case, then dose reduction may be sufficient in ameliorating HN (Kim et al. 2014).

### Risk factors for HN

The occurrence of drug-induced HN is linked to a variety of risk factors. Concomitant use of DIUs (Siegler et al. 1995; Kirby and Ames 2001; Roxanas 2003; Letmaier et al. 2012; Mannesse et al. 2013; De Picker et al. 2014), age ≥ 65 years (Movig et al. 2002; Wright and Schroeter 2008; Letmaier et al. 2012; De Picker et al. 2014), and female sex (Roxanas 2003; Wright and Schroeter 2008; Letmaier et al. 2012; Ramírez et al. 2019) are several of the most commonly reported risk factors of HN. Other studies were unable to identify an association with sex (Movig et al. 2002; Mannesse et al. 2013).

While often considered a safer alternative in the treatment of older adults due to their lower potential of causing antimuscarinic ADRs, patient’s ≥ 65 years of age may have a more than the sixfold increased risk in comparison to patients < 65 years of age of developing SSRI-induced HN (Movig et al. 2002). This study was able to specifically pinpoint a significantly higher risk of HN in female SSNRI-users aged ≥ 65 years, who were concomitantly treated with other potentially HN-inducing drugs. Among diagnostic subgroups, incidence of HN was highest among patients with substance-related disorders, which was also considered a predisposing factor in several cases of severe symptomatic HN. Substance abuse—in particularly of alcohol—is linked with the occurrence of HN most commonly due to hypovolemia (Liamis et al. 2000). Previously reported risk factors of HN, some of which this study was unable to evaluate due to missing information, include a history of cancer (Bourgeois 2005), previous HN (Fabian et al. 2004), lower BMI, and higher outdoor temperatures (Ramírez et al. 2019).

## Strengths and limitations

AMSP is a structured drug surveillance program with a uniform documentation process. The 23-year observation period of nearly half a million psychiatric inpatients enables the detection of rare ADRs with a lower margin of error. Due to the inpatient setting, AMSP is able to assess actual drug utilization rates versus prescription rates, as is often the case in studies reflecting the outpatient setting. All (suspected) ADRs are rigorously analyzed and reviewed by the drug monitors, senior physicians, and by the board before they are admitted into the AMSP database to counteract possible differences in individual judgement and assessment habits.

However, the present study should also be interpreted in the context of its limitations. Depending on time, motivation, and the financial means of the participating hospital, an individual and institutional bias in terms of underreporting of ADRs cannot be excluded. Patients receiving certain drugs with a more well-known risk of HN such as oxcarbazepine and carbamazepine may have been more closely monitored for the occurrence of HN than patients treated with drugs rarely associated with HN. However, it is expected that this collective of inpatients is more closely monitored for changes in laboratory values than outpatients due to routine blood collection. As most cases of HN in this study presented without symptoms or unspecific symptoms, that may be mistaken for a worsening of mental illness, complicating detection of HN in this specific study population. Drug monitors documenting the ADRs were often not directly involved in the treatment of the patients, therefore the description of ADRs could only be provided by the treating physician or their documentation. This may have resulted in a larger quantity of asymptomatic cases of HN. Due to AMSP’s strict criteria for inclusion of drug-induced HN in its database, the present study detected rates of HN much lower than other authors. While on the one hand this thorough screening and evaluation of ADR cases is beneficial and more accurately reflects the actual clinical relevance of ADRs, it also contributes to under-reporting. Furthermore, the results presented here may not represent the risk of HN in the ambulatory setting. Psychiatric inpatients are generally more severely ill and may also suffer from a higher degree of comorbidity, therefore potentially resulting in higher concomitant use of psychotropic and non-psychotropic drugs.

## Conclusion

As revealed in the present study, HN is a potential ADR of most antidepressant and antipsychotic drugs and is most likely to occur within the first weeks of treatment. While cases often presented without any clinical symptoms, severe symptoms such as seizures, coma, and delirium are potentially life-threatening and can result in permanent damage. The risk of HN varied among the various classes of psychotropic drugs. AEDs—especially carbamazepine and oxcarbazepine—were most likely to cause drug-induced HN. Among ADDs, SSRI and SSNRIs presented the highest risk of HN, while NaSSAs and TCAs were only rarely associated with HN. In comparison to ADDs as a group, APDs showed a much lower risk of HN. The risk of HN under treatment with SSRIs, SSNRIs, and carbamazepine increased significantly when combined with other potentially HN-inducing drugs used in the treatment of internal illnesses such as DIUs, ACE-Is, ARBs, and PPIs. This study found that women aged 65 years and older treated with SSNRIs with concomitant drug use had a particularly high risk of developing drug-induced HN. The identification of specific at-risk patient groups and high-risk drug combinations facilitates the implementation of drug safety in a clinically relevant manner.

## References

[CR1] Ajlouni K, Kern MW, Tures JF, Theil GB, Hagen TC (1974). Thiothixene-induced hyponatremia. Arch Intern Med.

[CR2] Bahar MA, Kamp J, Borgsteede SD, Hak E, Wilffert B (2018). The impact of CYP2D6 mediated drug–drug interaction: a systematic review on a combination of metoprolol and paroxetine/fluoxetine. Br J Clin Pharmacol.

[CR3] Bourgeois JA (2005). Reversible hyponatremia and venlafaxine. Psychosomatics.

[CR4] Bullmann C (2016). Hyponatriämie. Arzneimittelverordnung in Der Praxis.

[CR5] Coupland C, Dhiman P, Morriss R, Arthur A, Barton G, Hippisley-Cox J (2011). Antidepressant use and risk of adverse outcomes in older people: population based cohort study. BMJ.

[CR6] Coupland CA, Dhiman P, Barton G, Morriss R, Arthur A, Sach T, Hippisley-Cox J (2011). A study of the safety and harms of antidepressant drugs for older people: a cohort study using a large primary care database. Health Technol Assess.

[CR7] De Picker L, Van Den Eede F, Dumont G, Moorkens G, Sabbe BG (2014). Antidepressants and the risk of hyponatremia: a class-by-class review of literature. Psychosomatics.

[CR8] Degner D, Grohmann R, Kropp S, Rüther E, Bender S, Engel RR, Schmidt LG (2004). Severe adverse drug reactions of antidepressants: results of the German multicenter drug surveillance program AMSP. Pharmacopsychiatry.

[CR9] Dineen R, Thompson CJ, Sherlock M (2017). Hyponatraemia—presentations and management. Clin Med.

[CR10] Druschky K, Bleich S, Grohmann R, Engel RR, Kleimann A, Stübner S, Greil W, Toto S (2018). Use and safety of antiepileptic drugs in psychiatric inpatients-data from the AMSP study. Eur Arch Psychiatry Clin Neurosci.

[CR11] Dundas B, Harris M, Narasimhan M (2007). Psychogenic polydipsia review: etiology, differential, and treatment. Curr Psychiatry Rep.

[CR12] Egger C, Muehlbacher M, Nickel M, Geretsegger C, Stuppaeck C (2006). A review on hyponatremia associated with SSRIs, reboxetine and venlafaxine. Int J Psychiatry Clin Pract.

[CR13] Fabian TJ, Amico JA, Kroboth PD, Mulsant BH, Corey SE, Begley AE, Bensasi SG, Weber E, Dew MA, Reynolds CF, Pollock BG (2004). Paroxetine-induced hyponatremia in older adults: a 12-week prospective study. Arch Intern Med.

[CR14] Falhammar H, Lindh JD, Calissendorff J, Farmand S, Skov J, Nathanson D, Mannheimer B (2018). Differences in associations of antiepileptic drugs and hospitalization due to hyponatremia: a population-based case-control study. Seizure.

[CR15] Falhammar H, Lindh JD, Calissendorff J, Skov J, Nathanson D, Mannheimer B (2019). Antipsychotics and severe hyponatremia: a Swedish population-based case-control study. Eur J Intern Med.

[CR16] Falhammar H, Lindh JD, Calissendorff J, Skov J, Nathanson D, Mannheimer B (2019). Associations of proton pump inhibitors and hospitalization due to hyponatremia: a population–based case–control study. Eur J Intern Med.

[CR17] Falhammar H, Skov J, Calissendorff J, Nathanson D, Lindh JD, Mannheimer B (2020). Associations Between antihypertensive medications and severe hyponatremia: a Swedish population–based case–control study. J Clin Endocrinol Metab.

[CR18] Farmand S, Lindh JD, Calissendorff J, Skov J, Falhammar H, Nathanson D, Mannheimer B (2018). Differences in associations of antidepressants and hospitalization due to hyponatremia. Am J Med.

[CR19] Ferguson JM, Hill H (2006). Pharmacokinetics of fluoxetine in elderly men and women. Gerontology.

[CR20] Ferreira F, Mateus S, Santos AR, Moreira H, Ferreira NR (2016). Pantoprazole-related Symptomatic Hyponatremia. Eur J Case Rep Intern Med.

[CR21] Fort E, Laurin C, Baroudi A, Liebaert-Bories MP, Strock P (2000). Lansoprazole-induced hyponatremia. Gastroenterol Clin Biol.

[CR22] Gandhi S, McArthur E, Reiss JP, Mamdani MM, Hackam DG, Weir MA, Garg AX (2016). Atypical antipsychotic medications and hyponatremia in older adults: a population-based cohort study. Can J Kidney Health Dis.

[CR23] Gankam Kengne F, Andres C, Sattar L, Melot C, Decaux G (2008). Mild hyponatremia and risk of fracture in the ambulatory elderly. QJM.

[CR24] Gankam-Kengne F, Ayers C, Khera A, de Lemos J, Maalouf NM (2013). Mild hyponatremia is associated with an increased risk of death in an ambulatory setting. Kidney Int.

[CR25] Grohmann R, Engel RR, Rüther E, Hippius H (2004). The AMSP drug safety program: methods and global results. Pharmacopsychiatry.

[CR26] Grohmann R, Engel RR, Möller HJ, Rüther E, van der Velden JW, Stübner S (2014). Flupentixol use and adverse reactions in comparison with other common first- and second-generation antipsychotics: data from the AMSP study. Eur Arch Psychiatry Clin Neurosci.

[CR27] Holtschmidt-Täschner B, Soyka M (2007). Hyponatremia-induced seizure during carbamazepine treatment. World J Biol Psychiatry.

[CR28] Hwang AS, Magraw RM (1989). Syndrome of inappropriate secretion of antidiuretic hormone due to fluoxetine. Am J Psychiatry.

[CR29] Intravooth T, Staack AM, Juerges K, Stockinger J, Steinhoff BJ (2018). Antiepileptic drugs-induced hyponatremia: review and analysis of 560 hospitalized patients. Epilepsy Res.

[CR30] Jacob S, Spinler SA (2006). Hyponatremia associated with selective serotonin-reuptake inhibitors in older adults. Ann Pharmacother.

[CR31] Kim YS, Kim DW, Jung KH, Lee ST, Kang BS, Byun JI, Yeom JS, Chu K, Lee SK (2014). Frequency of and risk factors for oxcarbazepine-induced severe and symptomatic hyponatremia. Seizure.

[CR32] Kirby D, Ames D (2001). Hyponatraemia and selective serotonin re-uptake inhibitors in elderly patients. Int J Geriatr Psychiatry.

[CR33] Knigge U, Willems E, Kjaer A, Jørgensen H, Warberg J (1999). Histaminergic and catecholaminergic interactions in the central regulation of vasopressin and oxytocin secretion. Endocrinology.

[CR34] Kuz GM, Manssourian A (2005). Carbamazepine-induced hyponatremia: assessment of risk factors. Ann Pharmacother.

[CR35] Letmaier M, Painold A, Holl AK, Vergin H, Engel R, Konstantinidis A, Kasper S, Grohmann R (2012). Hyponatraemia during psychopharmacological treatment: results of a drug surveillance programme. Int J Neuropsychopharmacol.

[CR36] Liamis GL, Milionis HJ, Rizos EC, Siamopoulos KC, Elisaf MS (2000). Mechanisms of hyponatraemia in alcohol patients. Alcohol Alcohol.

[CR37] Liamis G, Milionis H, Elisaf M (2008). A review of drug-induced hyponatremia. Am J Kidney Dis.

[CR38] Lin CH, Lu CH, Wang FJ, Tsai MH, Chang WN, Tsai NW, Lai SL, Tseng YL, Chuang YC (2010). Risk factors of oxcarbazepine-induced hyponatremia in patients with epilepsy. Clin Neuropharmacol.

[CR39] Liu BA, Mittmann N, Knowles SR, Shear NH (1996). Hyponatremia and the syndrome of inappropriate secretion of antidiuretic hormone associated with the use of selective serotonin reuptake inhibitors: a review of spontaneous reports. CMAJ.

[CR40] Madhusoodanan S, Bogunovic OJ, Moise D, Brenner R, Markowitz S, Sotelo J (2002). Hyponatraemia associated with psychotropic medications. A review of the literature and spontaneous reports. Adverse Drug React Toxicol Rev.

[CR41] Mannesse CK, Jansen PA, Van Marum RJ, Sival RC, Kok RM, Haffmans PM, Egberts TC (2013). Characteristics, prevalence, risk factors, and underlying mechanism of hyponatremia in elderly patients treated with antidepressants: a cross-sectional study. Maturitas.

[CR42] Mazhar F, Pozzi M, Gentili M, Scatigna M, Clementi E, Radice S, Carnovale C (2019). Association of hyponatraemia and antidepressant drugs: a pharmacovigilance-pharmacodynamic assessment through an analysis of the US Food and Drug Administration Adverse Event Reporting System (FAERS) Database. CNS Drugs.

[CR43] Mazhar F, Carnovale C, Haider N, Ahmed R, Taha M (2020). Paliperidone-associated hyponatremia: report of a fatal case with analysis of cases reported in the literature and to the US Food and Drug Administration Adverse Event Reporting System. J Clin Psychopharmacol.

[CR44] Mazhar F, Battini V, Pozzi M, Invernizzi E, Mosini G, Gringeri M, Capuano A, Scavone C, Radice S, Clementi E, Carnovale C (2021). Hyponatraemia following antipsychotic treatment: In-silico pharmacodynamics analysis of spontaneous reports from the US Food and Drug Administration Adverse Event Reporting System Database and an updated systematic review. Int J Neuropsychopharmacol.

[CR45] Meulendijks D, Mannesse CK, Jansen PA, van Marum RJ, Egberts TC (2010). Antipsychotic-induced hyponatraemia: a systematic review of the published evidence. Drug Saf.

[CR46] Movig KL, Leufkens HG, Lenderink AW, van den Akker VG, Hodiamont PP, Goldschmidt HM, Egberts AC (2002). Association between antidepressant drug use and hyponatraemia: a case–control study. Br J Clin Pharmacol.

[CR47] Nachnani J, Bulchandani D, Bulchandani S (2015). Severe hyponatremia associated with the use of pantoprazole. Am J Gastroenterol.

[CR48] Naharcý MI, Cintosun U, Ozturk A, Bozoglu E, Doruk H (2014). Pantoprazole sodium-induced hyponatremia in a frail elderly adult. J Am Geriatr Soc.

[CR49] Palmer S, Vecchio M, Craig JC, Tonelli M, Johnson DW, Nicolucci A, Pellegrini F, Saglimbene V, Logroscino G, Fishbane S, Strippoli GFM (2013). Prevalence of depression in chronic kidney disease: systematic review and meta-analysis of observational studies. Kidney Int.

[CR50] Peck V, Shenkman L (1979). Haloperidol-induced syndrome of inappropriate secretion of antidiuretic hormone. Clin Pharmacol Ther.

[CR51] Ramírez E, Rodríguez A, Queiruga J, García I, Díaz L, Martínez L, Muñoz R, Muñoz M, Tong HY, Martínez JC, Borobia AM, Carcas AJ, Frías J (2019). Severe hyponatremia is often drug induced: 10-year results of a prospective pharmacovigilance program. Clin Pharmacol Ther.

[CR52] Renneboog B, Musch W, Vandemergel X, Manto MU, Decaux G (2006). Mild chronic hyponatremia is associated with falls, unsteadiness, and attention deficits. Am J Med.

[CR53] Rosner MH (2004). Severe hyponatremia associated with the combined use of thiazide diuretics and selective serotonin reuptake inhibitors. Am J Med Sci.

[CR54] Roxanas MG (2003). Mirtazapine-induced hyponatraemia. Med J Aust.

[CR55] Shepshelovich D, Schechter A, Calvarysky B, Diker-Cohen T, Rozen-Zvi B, Gafter-Gvili A (2017). Medication-induced SIADH: distribution and characterization according to medication class. Br J Clin Pharmacol.

[CR56] Siegler EL, Tamres D, Berlin JA, Allen-Taylor L, Strom BL (1995). Risk factors for the development of hyponatremia in psychiatric inpatients. Arch Intern Med.

[CR57] Spasovski G, Vanholder R, Allolio B, Annane D, Ball S, Bichet D, Decaux G, Fenske W, Hoorn EJ, Ichai C, Joannidis M, Soupart A, Zietse R, Haller M, van der Veer S, Van Biesen W, Nagler E (2014). Clinical practice guideline on diagnosis and treatment of hyponatraemia. Nephrol Dial Transplant.

[CR58] Spigset O, Hedenmalm K (1995). Hyponatraemia and the syndrome of inappropriate antidiuretic hormone secretion (SIADH) induced by psychotropic drugs. Drug Saf.

[CR59] Stedman CA, Begg EJ, Kennedy MA, Roberts R, Wilkinson TJ (2002). Cytochrome P450 2D6 genotype does not predict SSRI (fluoxetine or paroxetine) induced hyponatraemia. Hum Psychopharmacol.

[CR60] Strachan J, Shepherd J (1998). Hyponatraemia associated with the use of selective serotonin re-uptake inhibitors. Aust N Z J Psychiatry.

[CR61] Van Amelsvoort T, Bakshi R, Devaux CB, Schwabe S (1994). Hyponatremia associated with carbamazepine and oxcarbazepine therapy: a review. Epilepsia.

[CR62] van der Zalm IJB, Tobé TJM, Logtenberg SJJ (2020). Omeprazole-induced and pantoprazole-induced asymptomatic hyponatremia: a case report. J Med Case Reports.

[CR63] Verbalis JG, Barsony J, Sugimura Y, Tian Y, Adams DJ, Carter EA, Resnick HE (2010). Hyponatremia-induced osteoporosis. J Bone Miner Res.

[CR64] Vollset SE (1993). Confidence intervals for a binomial proportion. Stat Med.

[CR65] Wilkinson TJ, Begg EJ, Winter AC, Sainsbury R (1999). Incidence and risk factors for hyponatraemia following treatment with fluoxetine or paroxetine in elderly people. Br J Clin Pharmacol.

[CR66] Wright SK, Schroeter S (2008). Hyponatremia as a complication of selective serotonin reuptake inhibitors. J Am Acad Nurse Pract.

[CR67] Yang HJ, Cheng WJ (2017). Antipsychotic use is a risk factor for hyponatremia in patients with schizophrenia: a 15-year follow-up study. Psychopharmacology.

[CR067] Yip TC, Wong GL, Tse YK, Yuen BW, Luk HW, Lam MH, Li MK, Loo CK, Tsang OT, Tsang SW, Chan HL, Wing YK, Wong VW. High incidence of hepatocellular carcinoma and cirrhotic complications in patients with psychiatric illness: a territory-wide cohort study. BMC Gastroenterol. 2020;20(1):128. 10.1186/s12876-020-01277-0. PMID: 32349708; PMCID: PMC7189713. 10.1186/s12876-020-01277-0 PMC7189713 32349708

